# Internal consistency of the Mental Health Professional Culture Inventory: A pilot study in Romanian population

**DOI:** 10.1515/tnsci-2022-0350

**Published:** 2024-12-05

**Authors:** Frédéric Denis, Hélène Kane, Jade Gourret Baumgart, Emmanuel Rusch, Jocelyn Deloyer, Claudio Fuenzalida, Gabriela Kelemen, Mihaela Gavrila-Ardelean, Marek Krzystanek, Donatella Marazziti, Margarita Moraitou, Merja Reunanen, Rexhaj Shyhrete, Wissam El Hage, Johannes Thome, Wim Verwaest, Nathalie Rude, Charline Laruppe, Laurence Fond-Harmant

**Affiliations:** EA 75-05 Laboratory of Education, Ethics, Health, Faculty of Medicine, François Rabelais University, 1 avenue de la république, Boulevard Tonnellé, Chambray-lès-Tours, 37170, Tours, France; St-Martin Neuro Psychiatric Center, Namur, Belgium; Intrafamily Therapy Center, Elche, Spain; Faculty of Education Sciences, Psychology and Social Sciences, Aurel Vlaicu University of Arad, Arad, Romania; Clinic of Psychiatric Rehabilitation, Faculty of Medical Sciences, Medical University of Silesia, Katowice, Poland; Department of Clinical and Experimental Medicine, University of Pisa, Pisa, Italy; Saint Camillus International, UniCamillus, University of Health Sciences and Medicine, Rome, Italy; Social and Educational Support Center, Kepsipi, Korydallos, Greece; Step-Education, Pieksämäki, Finland; School of Nursing Sciences, HES-SO University of Applied Sciences Western Switzerland, Lausanne, Switzerland; CHRU de Tours, UMR 1253, iBrain, Université de Tours, Inserm, EE 1901 Qualipsy, 37000, Tours, France; Psychiatry Department, Rostock University, Rostock, Germany; Neuro-Psychiatric Hospital Center of Luxembourg, Ettelbruck, Luxembourg; Laboratoire de Recherches Intégratives en Neurosciences et Psychologie Cognitive, UFR Santé, Université de Franche-Comté, LINC, F-25000, Besançon, France; Agency for Europe-Africa Scientific Cooperation, Luxembourg, Luxembourg; EA 3412 Education and Healthcare Practices Laboratories, Sorbonne Paris Nord University, Paris, France

**Keywords:** psychometric, psychiatry, Romania, mental health, professional culture

## Abstract

**Background:** The objective of this study (registered under number 2020 006) was to assess the internal consistency of the revised Mental Health Professional Culture Inventory (MHPCI) scale, which comprises 15 items, among mental health service workers in Romania.

**Methods:** To examine the psychometric properties of the MHPCI questionnaire within the Romanian population, we employed two main methods: The partial credit model (PCM) and Exploratory factor analysis (EFA).

**Results:** A total of 94 individuals were interviewed, and among them, 71 provided complete responses to the questionnaire. All 15 items demonstrate a strong fit with the PCM, as indicated by mean-square (MSQ) outfit and MSQ infit values falling within the range of 0.5 to 1.5. But items 3 and 11 exhibit MSQ values greater than 1.5, suggesting that it may be challenging to predict individuals’ responses to these items. The KMO index stands at 0.7, surpassing the recommended threshold of 0.6, signifying an acceptable level of suitability. Nevertheless, only 59.3% of the total variance is accounted for by the first four factors, and these factors do not align with the dimensions identified in the original article.

**Conclusion:** The internal structure of the Romanian version of the MHPCI demonstrates satisfactory psychometric properties. These properties will need to be further validated through additional studies conducted in diverse socio-cultural contexts.

## 1 Introduction

The social psychologist Maria Jahoda points out that “a healthy work environment is one where employees feel valued, supported and able to flourish without fear of stigma.” Consequently, professional culture aims to create a harmonious and productive working environment in which employees feel engaged and productive. It also serves as a reflection of a shared professional history that brings individuals together within a community of professions characterized by similar training and experience. It fosters, for example, a sense of belonging and solidarity among employees, which can improve collaboration and communication within a team.

In the realm of mental health, Rapisarda and Miglioretti conducted a study that identified three interrelated dimensions contributing to the professional culture of healthcare staff: “interpersonal distance from users,” “power dynamics,” and specific interprofessional distinctions [1]. It is worth noting that certain paternalistic attitudes, still prevalent in some institutions, contradict an approach that could facilitate patients’ reconnection with reality by enabling them to take action and make decisions for themselves. This process of empowerment aimed at increasing an individual’s autonomy and responsibility for their own recovery, raises intricate and multifaceted issues, notably necessitating a shift in the attitudes and practices of mental health professionals [2].

In other words, a recovery-oriented approach emphasizes that service delivery should be guided by the individual needs and preferences of service users rather than being driven solely by the priorities of organizations or staff. Current evidence strongly supports the importance of an empowerment-based approach in enhancing the health outcomes of individuals receiving mental health services [3,4,5,6]. However, the implementation of this practice has been slower than desired. Several factors contribute to this, including clinicians’ paternalistic attitudes, concerns about stigma, fear of legal repercussions, the cognitive effects of mental illness, and the internalization of self-stigma [7,8]. Additionally, recovery objectives and action plans often continue to be influenced by the values and perspectives of staff or the organization rather than aligning with the priorities of service users [9,10].

In this context, the Mental Health Professional Culture Inventory (MHPCI) is designed to investigate the concept of “professional culture” among mental health service providers. Its purpose is to raise awareness among these professionals regarding the necessity of updating their patient care practices by embracing new professional behaviors and fresh perspectives on mental health care [11]. The initial version of the MHPCI questionnaire consisted of 71 items, carefully chosen by a collaborative team of Canadian and Italian researchers actively engaged in community mental health care and deinstitutionalization efforts. These items were crafted to capture a spectrum of behaviors, emotions, and cognitive assessments that a staff member might encounter while working with service users, with a preference for items focusing on behavior rather than explicit beliefs. After conducting a study to assess the questionnaire’s internal consistency, stability over time, and cross-cultural validity between the Canadian and Italian versions, 15 items were ultimately selected. Responses to these questions were rated on a 5-point Likert scale [12], resulting in four key themes: (1) the therapeutic framework, (2) the management of aggression risk, (3) family involvement, and (4) users’ sexuality.

Romania, like any other country in the world, needs to find ways of improving the quality of its mental health services [13]. Improving the culture of mental health in the workplace is an important part of this process. The MHPCI questionnaire can contribute to a better understanding of how the professional culture of mental health in Romania is characterized.

The objective of this study was to assess the internal consistency of the revised MHPCI scale, which comprised 15 items, among mental health service workers in Romania.

## 2 Materials and methods

### 2.1 Research design

Responses to the MHPCI questionnaire were collected as part of the Psy-GIPOC study, which is part of a collaborative effort aimed at analyzing how professionals working in psychiatric and mental health services were impacted by the pandemic [14]. This was a major international survey of 2,000 professionals in 23 countries over a 12-month period. This mixed qualitative and quantitative survey included 30 semi-structured individual interviews and 20 focus group sessions, as well as a digital questionnaire sent online to 2,000 psychiatric and mental health professionals. The aim of the Psy-GIPOC study was to take stock of the quality of existing structures and the availability of emergency structures with human resources in good physical and mental health and to draw lessons for developing effective processes for preparing for and anticipating crises [15,16]. Responses to the MHPCI questionnaire were collected and anonymized between March and November 2021. Participants were selected by ensuring that they met the study criteria, and to avoid multiple entries from the same participant each patient was clearly identified by a number. The questionnaires were configured in such a way that responses were collected without identifying information, and participants were informed at the beginning of the questionnaire of the measures taken to protect their data. As part of an ancillary study to the Psy-GIPOC study, the internal structure of this questionnaire was studied in the Romanian respondents to the study.

The MHPCI questionnaire consisted of 59 questions, comprising 43 close-ended and 16 open-ended questions, which included the 15 MHPCI questions. It was administered using Sphinx©. Prior to distribution, the final version of the questionnaire was rigorously tested and validated in the language of each country where the study was conducted.

The members of the research consortium shared the questionnaire link online from July to November 2021, using their professional and institutional social networks for a snowball sampling approach. The questionnaire was primarily directed toward professional associations in the field of psychiatry and mental health, as well as associations involved in related activities.

To ensure consistency in responses, the scale items were recorded in a manner that encouraged positive responses for all affirmations.

### 2.2 Analysis

**Figure 1:**
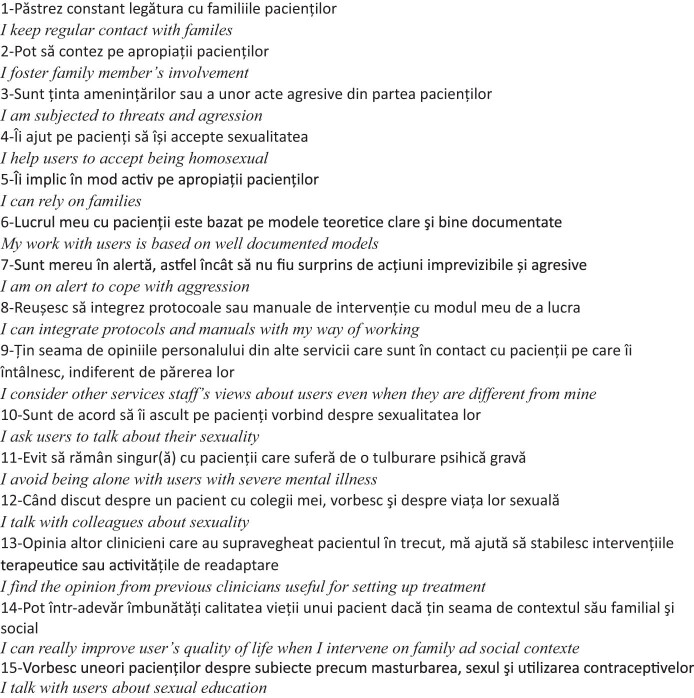
The 15 items of the MHPCI scale (Romanian version).

To examine the psychometric properties of the MHPCI questionnaire (15 items) within the Romanian population, as illustrated in Figure 1, we employed two main methods: the partial credit model (PCM) and exploratory factor analysis (EFA).

#### 2.2.1 Rasch analysis: PCM

The PCM is a unidimensional model designed for analyzing responses that are recorded in two or more ordered categories [17,18]. In this model, each item possesses its unique rating scale structure. It originally stems from multiple-choice tests, wherein responses that are incorrect but still indicate some degree of knowledge receive partial credit toward a correct response. The extent of partial correctness can vary from one item to another. Importantly, it permits the establishment of different thresholds for different items and shares an identical algebraic form with the Rasch model, albeit originating from a different starting point at a later stage [19].

One of the noteworthy features of the PCM is its capacity to facilitate “specifically objective” comparisons between individuals and items. Furthermore, it enables the conditioning of each set of model parameters out of the estimation procedure for the other [17].

#### 2.2.2 EFA

EFA is a statistical technique used to identify the underlying relationships between measured variables by calculating the variance-covariance matrix or correlation matrix of the selected variables. EFA is a multivariate statistical method frequently used in quantitative research and has begun to be used in many fields such as social sciences, health sciences, and economics. With EFA, researchers focus on fewer items that explain the structure, instead of considering too many items that may be unimportant and carry out their studies by placing these items into meaningful categories (factors). Importantly, EFA is conducted without making any assumptions about the number or structure of the instrument in question [20,21]. It is often used in conjunction with Rasch analysis [22]. The variance–covariance matrix or correlation matrix forms the basis for identifying the underlying factors. Examination of the factor loadings enables us to understand which variables are associated with each factor. Factor loadings closer to 1 or −1 indicate a strong relationship with the factor. Orthogonal rotation (e.g., Varimax) assumes that the factors are uncorrelated.

The primary objective of this analysis is to determine whether the correlations between items support the notion that these items are measuring the same underlying trait. If the items do not exhibit unidimensionality, it may be necessary to consider subdividing the scale into subscales or potentially eliminating certain items. This determination is typically based on criteria such as a Kaiser–Meyer–Olkin (KMO) value exceeding 0.6 and the Bartlett test yielding significant results indicating sphericity [20,21,22].

### 2.3 Sample

Evaluation of the MHPCI questionnaire, consisting of 15 items, was carried out among mental health professionals in Romania. The study applied the following inclusion criteria: staff members working in units or services dedicated to providing mental health care to individuals with severe mental illnesses, including those engaged in traineeships or voluntary work; staff members who have direct face-to-face contact with service users during their working hours; staff members representing a diverse range of mental health settings, whether within public or private organizations.

The anticipated number of participants was determined using G*Power R (wp.logistic) [22], a sample size calculation tool based on Cohen’s sampling formula. The parameters were set at a significance level of 0.05, a power of 0.80, and an effect size of 0.30, resulting in a target sample size of 75 individuals.

### 2.4 Data analysis

The analysis was conducted using the software package R^®^.


**Ethical approval:** The research related to human use complied with all the relevant national regulations and institutional policies, performed in accordance with the tenets of the Helsinki Declaration, and was approved by the authors’ institutional review board or equivalent committee. This research received ethical approval from the Ethics Committee of the Centre Hospitalier Régional Universitaire de Tours on February 4, 2021 (N° 2021 006). The authors confirm that all procedures in the study that involved participants comply with the ethical standards of the relevant national and institutional committees on human experimentation and with the Helsinki Declaration of 1964 and its later amendments.
**Informed consent:** Informed consent has been obtained from all individuals included in this study.

## 3 Results

### 3.1 General characteristics of Romanian participants

A total of 94 individuals were interviewed, and among them 71 provided complete responses to the questionnaire, representing a response rate of 75.5%. Details regarding the population’s characteristics can be found in the table below (Table 1).

**Table 1:** General characteristics of Romanian participants (*n* = 71)

Variables *N* (%)	
Gender (male)	10 (14.08)
Profession	
Psychiatric nursing assistant	1 (1.4)
Art therapist	1 (1.4)
Social worker	16 (22.5)
Psychiatric nurse	2 (2.8)
Physiotherapist	2 (2.8)
Dentist	1 (1.4)
General practitioner	2 (2.8)
Speech therapist	1 (1.4)
Psychiatrist	2 (2.8)
Psychologist	31 (43.7)
Healthcare executive	12 (16.9)
Private practice	21 (29.6)
Practice in hospitals	33 (46.5)
Combined experience	17 (23.9)
Years of experience	
21 ≤ 30	15 (21, 1)
≥30	3 (4, 2)
11 ≤ 20	17 (23, 9)
5 ≤ 10	36 (50, 7)

The sample size was in line with the sample calculated *a priori*, since with 71 people providing complete answers, all the analyses could be carried out in a statistically satisfactory manner. This confirms the general rule that between 5 and 10 participants per variable are needed to carry out an EFA [23].

### 3.2 Psychometric properties of the MHPCI questionnaire (15 items)

#### 3.2.1 Rasch analysis: PCM

The PCM specifies that each item has its own unique rating scale structure. It is derived from multiple-choice tests, wherein responses that are incorrect but indicate some level of knowledge are awarded partial credit toward a correct response. The degree of partial correctness varies among items (Table 2).

**Table 2:** PCM

	Location	Threshold 1	Threshold 2	Threshold 3	Threshold 4
Item 1	−0.15141	0.31515	−2.22191	0.66708	0.63402
Item 2	0.35234	0.061181	−1.78083	1.47133	1.65706
Item 3	−0.96755	−0.93934	−0.99576	NA	NA
Item 4	0.69184	3.16460	−0.80734	−0.28997	0.70007
Item 5	0.76641	−0.53236	−0.75438	1.07447	3.22789
Item 6	−0.87119	−0.37277	−2.62756	−0.74655	0.26212
Item 7	0.28166	−0.51588	0.16644	0.05213	1.42396
Item 8	−0.15471	−0.19192	−1.05162	−0.00643	0.63114
Item 9	−0.38551	−0.56028	−1.41355	−0.43765	0.86946
Item 10	−0.33350	0.83326	−2.03744	−0.05020	−0.07961
Item 11	0.92279	0.90785	1.49697	1.57517	−0.28884
Item 12	1.32207	1.05617	0.19920	3.39401	0.66892
Item 13	−0.19304	0.06062	−2.06047	0.10891	1.11877
Item 14	−0.52789	0.78023	−2.90951	−0.15009	0.16781
Item 15	0.77778	0.56449	0.08868	0.99537	1.46258

In the MHPCI questionnaire, all 15 items demonstrate a strong fit with the PCM, as indicated by the mean-square (MSQ) outfit and MSQ infit values falling within the range of 0.5 to 1.5. However, it is worth noting that items 3 and 11 exhibit MSQ values greater than 1.5, suggesting that it may be challenging to predict individuals’ responses to these particular items (Table 3).

**Table 3:** Fit statistics for the 15 items

	Outfit MSQ	Infit MSQ
Item 1	1.024	1.065
Item 2	0.971	1.002
Item 3	1.534	1.428
Item 4	1.276	1.000
Item 5	0.643	0.637
Item 6	0.730	0.775
Item 7	0.877	0.917
Item 8	0.845	0.849
Item 9	0.974	1.004
Item 10	0.964	0.965
Item 11	1.720	1.307
Item 12	0.986	0.897
Item 13	0.804	0.834
Item 14	0.791	0.767
Item 15	0.972	0.786

#### 3.2.2 EFA

**Figure 2:**
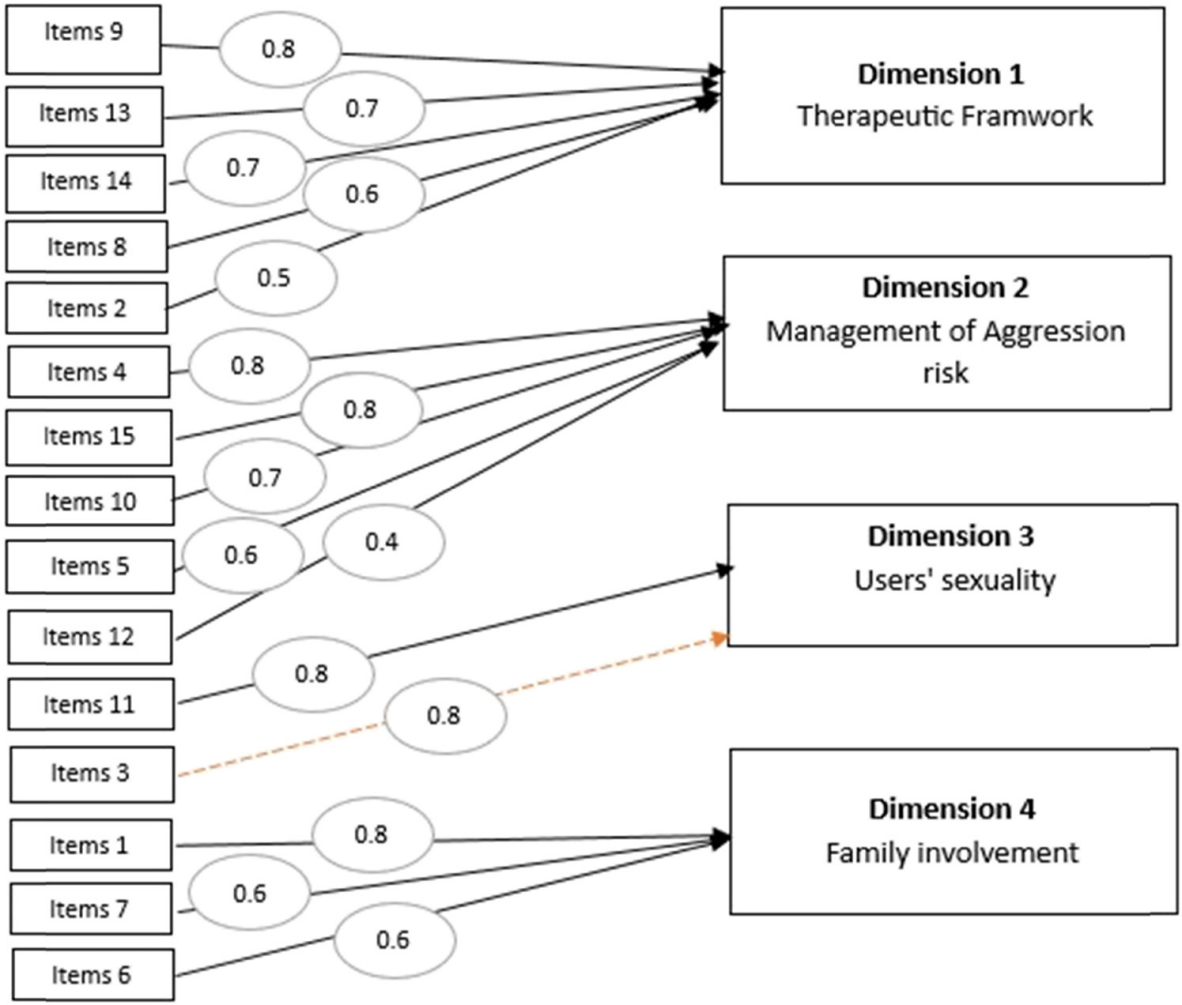
PCA.

The KMO index stands at 0.7, surpassing the recommended threshold of 0.6, signifying an acceptable level of suitability for principal component analysis (PCA). Nevertheless, it is noteworthy that only 59.3% of the total variance is accounted for by the first four factors, and these factors do not align with the dimensions identified in the original article. Additionally, item 3, “I am subjected to threats and aggression,” was omitted from the analysis (Table 4 and Figure 2).

**Table 4:** ACP Varimax MHPCI questionnaire (15 items)

	Dimension 1	Dimension 2	Dimension 3	Dimension 4
Item 2	0.48446037			
Item 8	0.56947744			
Item 14	0.68803951			
Item 13	0.71896878			
Item 9	0.79084796			
Item 11		0.80195963		
Item 12			0.42879206	
Item 5			0.5586002	
Item 10			0.71289667	
Item 15			0.79785817	
Item 4			0.80228674	
Item 6				0.62345419
Item 7				0.63627179
Item 1				0.77763728
Item 3				

## 4 Discussion

A study on the internal consistency of the Romanian version of the MHPCI scale (15 items) involving Romanian mental health workers demonstrated favorable psychometric properties related to its internal structure. However, disparities were observed in the categorization of items into different dimensions of the scale when compared to the original version. During the translation process with the mental health workers in Romania, no problems of this type were raised. This is in line with the fact that the Romanian population has comparable knowledge about mental illness to the American population [24] and a similar pattern of preferences regarding treatment, with both countries opting rather for non-biological, non-specialized treatment. Romanian mental health service users diagnosed with a first episode of mental illness reported less perceived and experienced stigma compared to their correspondents in Poland and Sweden [25].

In light of the findings in dimension 1, titled “Therapeutic Framework,” it appears that Romanian caregivers who participated in the study are committed to diligently applying the latest care recommendations for their patients. However, mental health services are underfunded, resulting in inadequate facilities and support systems. Research conducted a few years ago revealed that the Romanian mental health care system had 86% fewer social workers than officially required. The professionals highlighted damning facts, revealing that the skills of psychological workers are inadequate. They also mentioned that social workers receive inadequate pay and training. There is also a shortage of psychologists, with some 60% fewer than required [26]. Over a decade after the fall of Romanian communism, psychiatrists raised concerns about the increasing suicide rates and the prevalence of mental health disorders in the population [27,28]. It was within this context that the Council of Europe initiated a 2-year plan to bolster mental health care in Romania, especially in prisons, by increasing the number of medical staff [29,30]. This plan commenced in January 2022 and is backed by substantial financial support until the end of 2023, aimed at retaining psychiatrists in the country and offering them opportunities for ongoing professional and personal development [31].

Regarding the theme “Users’ Sexuality,” in the original version, this dimension comprises four items, but in the Romanian version, only item 11 is included [1]. The desire for affective and/or sexual relations, a universal experience, may not be freely expressed due to the influence of societal norms and values, which shape its approach. Furthermore, this experience may be restricted or suppressed by certain laws. For decades in Romania, all consensual sexual relations between adults of the same sex were prohibited [32]. In this context, it is likely why items from the “User’s Sexuality” dimension in the original version are placed within the “Management of Aggression Risk” dimension in the Romanian version (items 4, 15, 10, and 12).

Variations in item grouping also exist within the “Family Involvement” dimension, which may be linked to the specific characteristics and values of Romanian families. In Romanian culture, the age of marriage is typically earlier than in Western European countries, and celibacy or consensual cohabitation is an exception or the only concern of the transitional period before marriage. Additionally, the one-child family model appears to be increasingly common due to improved financial resources and living conditions, while relationships with the family of origin remain close [33]. More specifically, differences between generations were observed for individualistic and collectivist values, and in particular for self-construction. These differences were also apparent within families. These results confirm the social change that has taken place in post-communist Romania. Social change seems to have affected generations of parents and children differently, and in this context psychological characteristics are important resources that lead to a reduction in poverty and less negative perceptions of social change [34,35].

## 5 Implications for practice

Mental health professionals have been identified not only as a priority target for anti-stigma reduction but also as agents in the fight against stigma [36,37]. At present, training in anti-stigma advocacy is not part of the general training of mental health professionals or medical students in Romania. Research and educational fields need to work together to build and implement evidence-based actions to ensure educational and legal frameworks. The Romanian version of the MHPCI scale is a tool for mental health professionals and students to develop their practice in the future.

## 6 Limitations

The questionnaire was translated into Romanian and reviewed by the psychiatrists involved in the study. However, it was not back-translated into the original language to ensure that the Romanian version remained faithful to the original [38]. There is a possibility that a backtranslation could have introduced slight modifications to the Romanian version we obtained.

The survey was conducted during the COVID-19 pandemic, a period when healthcare staff were actively engaged in the field to provide support to patients [39]. It is conceivable that an emotional bias, influenced by the shock of the Covid crisis, may have influenced respondents’ choices. This emotional bias could be attributed not only to the impact of the crisis but also to the time required for individuals to step back and critically analyze their practices.

For all these reasons, our sample may not adequately represent the diverse population of mental health professionals in Romania, as several studies have highlighted an alarming level of stigma among health or mental health professionals [40,41]. Thus, although mental health professionals have a better knowledge of mental illness than other health specialists, they are predisposed to negative cultural stereotypes. Up to 50% of the mental health service users have reported discriminatory behavior on the part of mental health specialists [42,43].

The snowball sampling approach is a limitation of our study. This limitation is related to the lack of participants available to take part in the study. In the context of the pandemic, it was difficult to recruit mental health professionals in Romania. Many of them were focused on treating patients. However, as a form of convenience sampling based on a network, it can be seen as negative because it does not produce samples that meet the criteria of random sampling in the statistical sense (i.e., snowball samples deviate from probability-based sampling approaches). In other words, the dominant characteristic of the snowball sample depends on selection bias as well as a lack of external validity, generalizability, and representativeness. Furthermore, as Amanda Wilmot pointed out, a research sample of people who know each other may not only have similar views and experiences but also influence each other’s responses to interview questions [44].

## 7 Conclusions

The internal structure of the Romanian version of MHPCI demonstrates satisfactory psychometric properties. Nevertheless, it is worth noting that some items do not align with the dimensions of the original version. These disparities will need to be further validated through additional studies conducted in diverse socio-cultural contexts. Additionally, it is essential to explore the discriminant validity of this scale, possibly in comparison with other measures, such as the General Health Questionnaire scale, as described by Rapisarda et al., who authored the original MHPCI questionnaire.
